# Effect of Heart Rate Variability Biofeedback Sessions With Resonant Frequency Breathing on Sleep: A Pilot Study Among Family Caregivers of Patients With Cancer

**DOI:** 10.3389/fmed.2020.00061

**Published:** 2020-02-25

**Authors:** Hideaki Hasuo, Kenji Kanbara, Mikihiko Fukunaga

**Affiliations:** Department of Psychosomatic Medicine, Kansai Medical University, Osaka, Japan

**Keywords:** sleep, insomnia disorder, family caregiver, self-control, heart rate variability biofeedback, resonant frequency breathing

## Abstract

Heart rate variability biofeedback (HRV-BF) is used as a skill in psychosomatic medicine, but is not yet established in the field of sleep. The present study aimed to evaluate the effect of HRV-BF with resonant frequency breathing (RFB) on sleep performed once every 2 weeks and the usefulness of practice of RFB using a portable device at home before bedtime. Participants were 69 family caregivers of patients with cancer that felt burdened by nursing care. We conducted a randomized controlled trial with an HRV-BF+Home practice group and an HRV-BF group. HRV-BF with RFB was administered to both groups at our medical institution for up to 30 min on the experiment days. Home practice involved RFB using a portable device, which was performed at home each day within 20 min before bedtime. Evaluation items were: change ratio of total score of the Pittsburgh Sleep Quality Index (PSQI) at 28 days after the trial started. In total, 52.2% of participants had insomnia. The two HRV-BF groups had decreased PSQI total scores, which indicated an improvement in PSQI total score near 5.5 on Day 28. The two HRV-BF groups had significantly increased HRV scores on Day 28, and there was correlation between the variation of PSQI total score and the variation of HRV score. The quality of sleep assessed by PSQI scores in the HRV-BF+Home practice group was significantly improved compared with the HRV-BF group on Day 28 (*p* = 0.001). This suggests HRV-BF may be a useful skill for enhancing sleep among family caregivers of patients with cancer, as well as supporting their autonomic nervous function. Additional actual regular practice of RFB (using a portable device at home before bedtime) may further enhance the effect.

## Introduction

Insomnia is a common phenomenon for those who provide informal care for someone living with a life-limiting condition. Insomnia is a typical psychiatric disorder, with a prevalence of 72% [as measured by the Japanese version of the Pittsburgh Sleep Quality Index (PSQI)] among family caregivers of patients with advanced cancer in Japan (1). Treatment for insomnia disorder is mainly medication therapy, but there are problems associated with long-term treatment such as decreased self-efficacy, dependency, and developing tolerance (2). In early palliative care, patients with cancer and family caregivers showed increased use of approach-oriented skills, which was associated with higher self-efficacy and quality of life (3).

Biofeedback (BF) is a behavioral therapy that regulates the mind and body by measuring physiological information of which people are normally unaware and visually feeding back this information, which creates greater self-awareness and fosters better skills (4). A protocol for heart rate variability (HRV) BF (HRV-BF) sessions using resonant frequency breathing (RFB) has been established (5) and found useful for people with hyperactive autonomic nervous function, mood disorders, and fibromyalgia (6–8). HRV measures the fluctuation in the interval between heartbeats and reflects autonomic nervous activity. RFB is a method of breathing that maximizes HRV by creating a resonance between breathing and the baroreceptor reflex (5). RFB was found to enhance baroreflex sensitivity among patients with chronic heart failure (9).

Physical functions rest during sleep and breathing and heart rates decrease. HRV-BF with RFB uses respiratory sinus arrhythmia to increase HRV (5). In respiratory sinus arrhythmia, the heart rate increases when breathing in, and decreases when breathing out. It is hypothesized that respiratory sinus arrhythmia is an intrinsic resting function of the cardiopulmonary system (10). Respiratory sinus arrhythmia for healthy persons is reported to increase during non-rapid eye movement sleep (11). Therefore, HRV-BF with RFB may influence resting function during sleep. A previous report found healthy persons could increase respiratory sinus arrhythmia during sleep by HRV-BF before bedtime (12). Moreover, a randomized controlled trial involving an HRV-BF group and a control group investigated the use of a portable device in terms of the first night effect for healthy persons (13). Polysomnography showed that although there was no improvement in sleep latency in the HRV-BF group the following day, the quality of sleep was enhanced. A previous case report suggested that HRV-BF with use of a portable device for 1 week after application could improve insomnia disorder (14). However, few research reports have investigated if HRV-BF before bedtime or the practice of RFB could have a medium- to short-term effect on sleep. A controlled before-and-after trial involving patients with posttraumatic stress disorder indicated that HRV-BF with a portable device before bedtime significantly reduced the Insomnia Severity Index 4 weeks after application (15).

To our knowledge, the effect of an HRV-BF session on sleep with RFB has not been reported. A previous study was conducted in which HRV-BF sessions with RFB were performed every 2 weeks for family caregivers of patients with cancer (16). That study showed that after two sessions of HRV-BF, the HRV score increased on Day 28. The effect of the stress burden of lowered autonomic nervous function may reduce respiratory sinus arrhythmia during sleep (17). Therefore, even a short session of HRV-BF may show a positive effect on sleep irrespective of HRV-BF before bedtime or regular practice of RFB. The present study aimed to evaluate the influence of HRV-BF on sleep among family caregivers with a care burden. We aimed to prove the hypothesis that HRV-BF sessions with RFB would have an effect on sleep, and daily practice of RFB before bedtime at home could enhance this effect.

## Materials and Methods

### Study Design

We administered HRV-BF with RFB to family caregivers of patients with advanced cancer. We conducted an open-label randomized controlled study involving 69 family caregivers of patients with advanced cancer. The family caregivers were allocated to an HRV-BF+Home practice group or an HRV-BF group with a computer-generated algorithm using minimization methods and a 1:1 allocation ratio, according to whether they performed the RFB method using a portable device at their home (home practice). The HRV-BF+Home practice group performed HRV-BF with RFB at our medical institution on Days 0, 14, and 28 from the start of the intervention. This group also performed RFB using a portable device at home each day before bedtime (home practice). The HRV-BF group performed HRV-BF with RFB at our medical institution on Days 0, 14, and 28, but did not perform the home practice.

### Ethics Statement

This study was approved by the ethics committee of Kansai Medical University, Japan (No. 2015660). Written informed consent was obtained from all study participants. The procedures performed in this study were in accordance with the 1964 Helsinki Declaration and approved by the Human Research Ethics Committee of our institution. This study was registered with the University hospital Medical Information Network Clinical Trials Registry (approval number: UMIN000021639) on March 27, 2016.

### Participants

**Figure 1:**
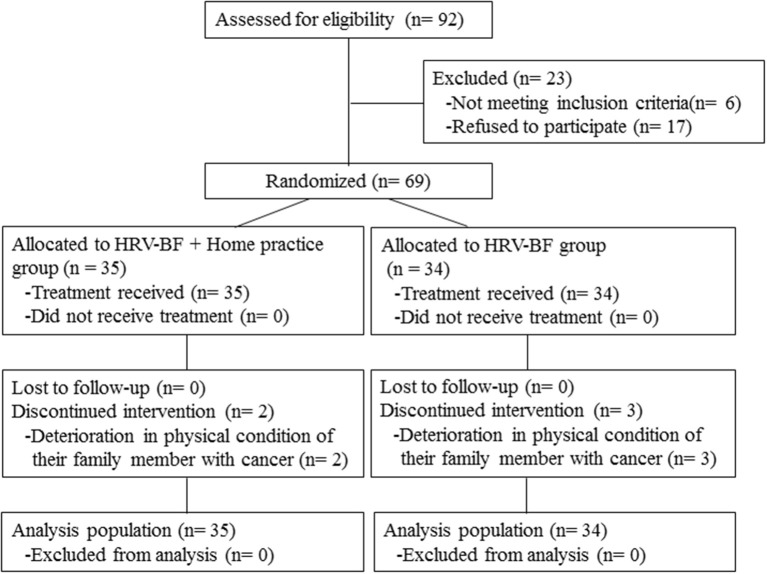
Study flow diagram. HRV, heart rate variability; BF, biofeedback.

This study was conducted from 2015 to 2018 at the Kansai Medical University Hospital. We evaluated the level of nursing-care burden experienced by family caregivers using the Japanese version of the Zarit Caregiver Burden Interview (J-ZBI). Participants with a J-ZBI score ≥24 (which is the cutoff value for depression risk) were eligible for this study (18). During this period, 244 family caregivers of patients with advanced cancer who visited the palliative care department were continuously enrolled in this study. Of these family caregivers, 92 met the study eligibility criteria according to their J-ZBI score. Exclusion criteria were: (1) having a disease that affected the evaluation of autonomic nerve function (e.g., diabetes); and (2) having any comorbidity relating to psychiatric disorders or conditions that made communication difficult (e.g., cognitive impairment). Figure 1 presents a flow diagram of this study.

### Measures

HRV-BF with RFB was administered to family caregivers of patients with advanced cancer at our medical institution for up to 30 min on Days 0, 14, and 28 from the start of the intervention. At Day 0, we monitored breathing among participants at our hospital using a multichannel biofeedback system (ProComp Infiniti™/BioGraph Infiniti; Thought Technology Ltd., Montreal, Canada) to determine participants' resonant frequency. Participants breathed for 2 min at 5, 5.5, 6, 6.5, and 7 breaths per minute while we measured resonant frequency. We calculated the RFB as the number of breaths that maximized the HRV spectrum peak and smoothed the HRV waveform. During HRV-BF, family caregivers used the Breath Pacer application (ProComp Infiniti™/BioGraph Infiniti; Thought Technology Ltd., Montreal, Canada) to maximize the HRV waveform displayed on the screen of a personal tablet computer (iPad mini; Apple, Cupertino, CA, USA). We connected the HRV components (myBeat WHS-2; Union Tool Co., Tokyo, Japan) to an electrode pad attached directly to the participant's chest. The HRV waveform was displayed on the screen in real time.

We downloaded the Breath Pacer application and a breath self-training guide onto portable devices for participants to use at home. Participants were instructed that home practice should be performed each day within 20 min (or a minimum of 5 min) before going to bed, according to the pace set by their portable device. Breath Pacer data entry was performed based on frequency after the user's RFB had been determined.

We measured HRV before administering HRV-BF. Family caregivers performed RFB for 5 min, resting for 5 min before and after RFB. We measured the HRV continuously for 15 min. These measurements were recorded on Days 0, 14, and 28. We used HRV analysis software (Kubios HRV version 3.1; Kubios Oy, Kuopio, Finland), which is considered highly reliable for short-term recording (19, 20). Furthermore, we evaluated family caregivers' sleep using the Japanese version of the PSQI (PSQI-J), which is a self-report questionnaire that we administered on Days 0 and 28. The data that support the findings of this study are openly available in figshare.

### Study Analytical Parameters

Analytical parameters included demographic factors, relationship with the patient, resonant frequency, and J-ZBI scores. We extracted additional information including rate of insomnia disorder, PSQI-J score, and HRV score. The primary outcomes of this study were the change rate in the total PSQI-J score and HRV score at 28 days after the intervention start, based on comparisons between the HRV-BF+Home practice group and the HRV-BF group.

#### Rate of Insomnia Disorder

We calculated the rate of insomnia disorder by dividing the family caregivers diagnosed with insomnia disorder by all family caregivers. Insomnia disorder was diagnosed when a family caregiver met all of the diagnostic criteria for insomnia in the Diagnostic and Statistical Manual of Mental Disorders, Fifth Edition (21).

#### PSQI-J

The PSQI-J is the most frequently used scale to assess insomnia occurring in the past month among family caregivers of patients with advanced cancer (1). The PSQI-J total score comprises seven component scores: sleep quality, sleep latency, sleep duration, habitual sleep efficiency, sleep difficulty, hypnotic use, and daytime dysfunction. Each component score ranges from 0 to 3. The PSQI-J total score is the total score of all components, with higher scores indicating poorer sleep. A PSQI-J global score cut off point of 5.5 yielded estimations of sensitivity and has been validated in caregivers of oncology patients, with a Cronbach's α of 0.68 (22).

#### HRV

HRV, which is the fluctuation of heartbeat interval, is used as a measure reflecting autonomic nerve activity. A low frequency (LF; 0.04–0.15 Hz) component and high frequency (HF; 0.15–0.4 Hz) component are recorded within several minutes of HRV (23). These components are obtained by frequency domain analysis and reflect parasympathetic activity. The standard deviation of the normal-to-normal interval (SDNN) is the standard deviation of the R-R intervals in an electrocardiogram, and is obtained through time domain analysis. A decrease in SDNN indicates a decrease in parasympathetic activity (24). The SDNN reflects all contributions to HRV, including sympathetic activity. The mean value for resting HRV in adults are LF = 519 ms^2^, HF = 657 ms^2^, and SDNN = 50 ms (25).

### Statistical Analysis

We used unpaired *t*-tests for the dependent variables: age, resonant frequency, J-ZBI scores, rate of insomnia disorder, PSQI-J total score (Day 0), and SDNN score (Day 0). We used Pearson's chi-square tests to analyze the dependent variables: sex, PSQI-J ≤5.5 or PSQI-J >5.5. Changes in the course of PSQI-J score and SDNN score (Days 0, 14, and 28) were analyzed using one-way repeated measures analysis of variance (ANOVA) for each group. To conduct comparisons between groups, we used time-course as the within-subjects factor and group as a between-subjects factor in two-way repeated measures ANOVA. In the ANOVA, multiple comparisons were corrected using the Bonferroni method. The main analysis was based on the intention-to-treat principle. If participants withdrew from the study, the PSQI-J scores and HRV scores after withdrawal were replaced with scores just before withdrawal. We defined withdrawal from home practice of RFB as missing practice more than twice a week. In addition, Pearson's correlation coefficients between the variation of PSQI-J total score and the variation of HRV score were calculated. A *p* < 0.05 was regarded as statistically significant. Statistical analyses were conducted using SPSS version 18.0J for Macintosh (SPSS, Inc. IBM, Chicago, IL).

## Results

Table 1 shows the demographic and clinical characteristics of the study groups. Insomnia disorder was diagnosed in 52.2% [95% confidence interval (CI): 40.2–64.2] of participants. No sleep-wake disorders other than insomnia disorder were observed. Family caregivers in the HRV-BF+Home practice group had a bigger burden of nursing care, and tended to have higher PSQI-J total scores and lower SDNN scores than the HRV-BF group; however, these differences were not statistically significant. Two family caregivers in the HRV-BF+Home practice group and three in the HRV-BF group withdrew from this study during Days 14–28 (completion rate 94.3 and 91.2%, respectively) because their family member with cancer experienced a decline in physical condition. There were no cases in which home practice of RFB was missed more than twice a week.

**Table 1:** Comparison of clinical characteristics between the HRV-BF + Home practice, HRV-BF, and control groups.

	**HRV-BF + Home practice group (*n* = 35)**		**HRV-BF group (*n* = 34)**		***P***
Age (years), mean (SD)	64.5	(10.5)	61.8	(12.6)	0.34
Sex, *n* (%)					
Male	12	(34)	11	(32)	0.87
Female	23	(66)	23	(68)	
Relationship with the patients, *n* (%)					
Mother	1	(2.9)	1	(2.9)	
Husband	11	(31.4)	10	(29.5)	
Wife	19	(54.2)	17	(50.0)	
Son	1	(2.9)	1	(2.9)	
Daughter	3	(8.6)	5	(14.7)	
Conditions of the patients with advanced cancer					
Outpatient, *n* (%)	32	(91.4)	30	(88.2)	0.67
Under cancer treatment, *n* (%)	24	(68.6)	25	(73.5)	0.94
Necessity of nighttime care, *n* (%)	5	(14.3)	3	(8.8)	0.49
Resonant frequency, mean (SD)	6.0	(0.7)	6.3	(0.7)	0.18
	***n***	**%**	***n***	**%**	
5	5	(14.3)	3	(8.8)	
5.5	9	(25.7)	6	(17.6)	
6	6	(17.1)	6	(17.6)	
6.5	8	(22.9)	8	(23.6)	
7	7	(20.0)	11	(32.4)	
J-ZBI, mean (SD)	37.7	(15.5)	35.1	(14.0)	0.46
Insomnia disorder, *n* (%)	19	(54.3)	17	(50.0)	0.73
PSQI-J score (day 0), mean (SD)	9.8	(3.0)	8.6	(3.9)	0.15
	***n***	**%**	***n***	**%**	
PSQI-J ≦ 5	4	(11.4)	4	(11.8)	0.97
PSQI-J > 5	31	(88.6)	30	(88.2)	
Heart rate variability, mean (SD)					
LF (day 0)	250.0	(116.7)	284.3.0	(178.9)	0.26
HF (day 0)	224.2	(107.4)	191.2	(118.5)	0.36
SDNN (day 0)	27.4	(7.2)	29.6	(9.4)	0.72

*HRV-BF, heart rate variability-biofeedback; J-ZBI, Japanese version of Zarit Caregiver Burden Interview; PSQI-J, Japanese version of the Pittsburgh Sleep Quality Index; LF: low frequency; SD, standard deviation*.

**Figure 2:**
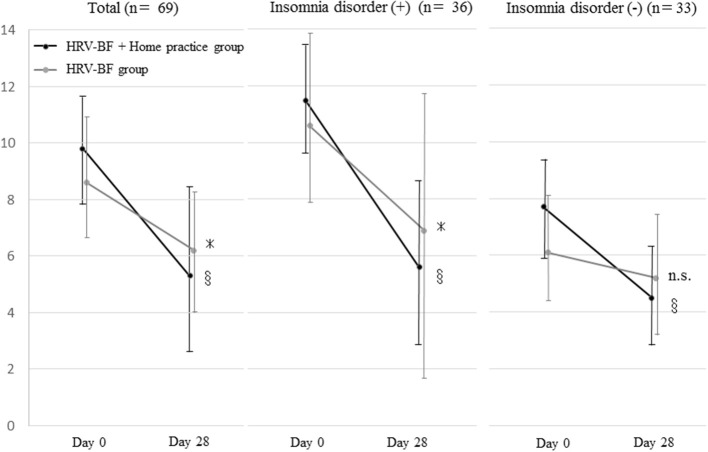
Change in the Japanese version of the Pittsburgh Sleep Quality Index total score. HRV, heart rate variability; BF, biofeedback; n.s., not significant. *p < 0.05 (vs. Day 0), ^§^*p* < 0.001 (vs. Day 0).

A comparison of the PSQI-J total scores between groups showed no significant interaction of time course and group (*p* = 0.114). There was only a main effect in time course (*p* < 0.001). The total PSQI-J scores of both groups were significantly decreased on Day 28 compared with Day 0. In family caregivers with and without insomnia disorder, there was no interaction between time course and group (*p* = 0.206 and *p* = 0.084, respectively). There was only a main effect in time course (*p* < 0.001 and *p* = 0.002, respectively) (Figure 2).

Most of the PSQI-J component scores of both groups were significantly decreased on Day 28 compared with Day 0. The sleep quality score showed a significant interaction of time course and group (*p* = 0.001). There was also a significant difference in time course between the groups (Day 0, *p* = 0.611; Day 28, *p* < 0.001). In the other component scores, the interactions of time course and group were not significant. In the HRV-BF+Home practice group, the score for sleep latency was increased, but there was no significant difference between before and after (Table 2).

**Table 2:** Change in the seven component scores of the Japanese version of the Pittsburgh Sleep Quality Index.

	**HRV-BF** **+** **Home practice group**			**HRV-BF group**			***P*^b^**
	**Day 0**	**Day 28**		**Day 0**	**Day 28**		
	**Mean (SD)**	**Mean (SD)**	***P^a^***	**Mean (SD)**	**Mean (SD)**	***P^a^***	
Sleep quality	19 (0.6)	0 5 (0.7)	<0.001	1.8 (0.8)	1.2 (0.8)	0.003	0.001
Sleep latency	13 (0.9)	1.5 (1.1)	0.560	1.1 (1.0)	1.0 (1.0)	0.817	0.683
Sleep duration	2.1 (0.8)	1.2 (1.1)	<0.001	1.8 (0.9)	1.4 (1.0)	0.109	0.093
Habitual sleep efficiency	1.3 (1.1)	0.4 (0.8)	<0.001	1.1 (1.2)	0.5 (1.0)	0.020	0.403
Sleep disturbances	1.0 (0.5)	0.5 (0.8)	0.001	0.8 (0.6)	0.8 (0.7)	0.852	0.062
Use of sleeping medication	0.5 (0.8)	0.3 (0.8)	0.305	0 5 (0.7)	0.4 (1.0)	0.891	0.554
Daytime dysfunction	1.6 (1.2)	0.8 (0.8)	0.001	1.6 (1.2)	0.7 (0.8)	0.001	0.811

a *unpaired t-test*,

b *two-way repeated measures analyses of variance. HRV, heart rate variability; BF, biofeedback*.

**Figure 3:**
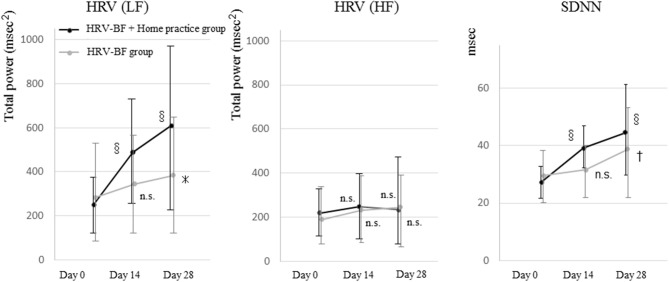
Change in heart rate variability before resonant breathing. HRV, heart rate variability; BF, biofeedback; SDNN, standard deviation of the normal-to-normal interval; LF, low frequency; HF, high frequency; n.s., not significant. *p < 0.05 (vs. Day 0), ^†^*p* < 0.01 (vs. Day 0), ^§^*p* < 0.001 (vs. Day 0).

The HRV scores of both groups, including LF and SDNN, were significantly increased on Day 28 compared with Day 0. There was a significant interaction between time course and group (*p* = 0.014) in LF between the groups. There was also a significant difference in time course between the groups (day 0, *p* = 0.571; day 14, *p* = 0.014; day 28, *p* = 0.001). When we compared SDNN between the groups, there was significant interaction between time course and group (*p* = 0.043), and a significant difference in time course between the groups (day 0, *p* = 0.453; day 14, *p* = 0.010; day 28, *p* = 0.045). We found no significant difference in HF between the groups, indicating that there was no interaction between time course and group (*p* = 0.923) (Figure 3).

The Pearson's correlation coefficient between the variation of PSQI-J total score and the variation of LF score was −0.365 (*p* = 0.007). The correlation coefficient between the variation of PSQI-J total score and the variation of SDNN score was −0.425 (*p* = 0.001). There was no correlation between the variation of PSQI-J total score and the variation of HF score (*p* = 0.484).

## Discussion

This study provided the first evaluation report that HRV-BF sessions conducted once every 2 weeks had an effect on sleep among family caregivers with a care burden, and a medium- to short-term effect on autonomic nervous function. In addition, daily practice of RFB at home enhanced this effect. This study targeted family caregivers that had not attended hospitals regularly, but who scored over 30 on the J-ZBI and reported a moderate level of nursing care burden (18). The level of distress among family caregivers of patients with advanced cancer as determined by the prevalence of insomnia disorder was extremely high (52.2%).

The study results highlighted two important points. First, there may be a positive effect on sleep even with only two sessions of HRV-BF, regardless of whether RFB was practiced at home before bedtime. The total PSQI-J scores in both groups decreased significantly, and showed an improvement in total PSQI-J score (near 5.5) on Day 28. This study had no control group (without HRV-BF), which was a limitation. However, the LF scores and SDNN scores in the two HRV-BF groups were significantly increased on Day 28, and there was a negative correlation between the variation in PSQI-J total scores and the variation in the LF scores and SDNN scores. It has been reported that LF scores in chronic insomnia patients is lowered during sleep, which is consistent with an increase in sympathetic activity before sleep (26). Therefore, the lowering of the parasympathetic nervous system may be a main pathogenic mechanism for primary insomnia disorder. This suggests that two sessions of HRV-BF had a positive effect on sleep through the interaction between sleep and autonomic nervous function. Previous studies involving HRV-BF sessions compared two time points (before and after the intervention) using 10 or 15 weekly HRV-BF sessions (6–8). Among those studies, one study involving 10 weekly sessions of HRV-BF among patients with depression compared pre-intervention and intervention using 4, 7, and 10 sessions. There was a significant decrease in the Depression Scale Score and an increase in SDNN after four sessions (Day 28) (7), which was similar to our results in terms of a rapidly produced effect. Our results are promising because they suggest that even one HRV-BF session may be feasible for improving skills of family caregivers of patients with cancer, regardless of whether RFB is practiced before bedtime.

The second important point in this study was that daily practice of RFB using a portable device at home enhanced the quality of both sleep and autonomic nervous function over a short period. In particular, the quality of sleep (evaluated by the PSQI-J scores) in the HRV-BF+Home practice group was significantly improved compared with the scores of those in the HRV-BF group on Day 28. Sleep quality was a major primary endpoint in previous studies (27, 28). A randomized controlled trial involving a HRV-BF group and a control group among 10 healthy persons with experience of polysomnography showed no improvement in sleep latency, but sleep quality was enhanced in the HRV-BF group (11). In addition, the HRV-BF+Home practice group showed a significant increase in LF and SDNN compared with that of the HRV-BF group as early as Day 14. RFB gives rise to a resonance between breathing and the baroreceptor reflex involving the LF power of HRV (but not HF power of HRV) to increase HRV (5). This suggests that continuous practice of RFB at home indicates a persistent effect, rather than only a transient effect at the time of application. Therefore, because RFB supports an efficient baroreceptor reflex, continuous practice may enhance autonomous homeostasis functions (5). Because there were few reports available for RFB with use of a portable device at home (29), we could not make comparisons with other tests. However, among the PSQI-J component scores, we found that only the numerical values for sleep latency became worse in the HRV-BF+Home practice group. It has been reported that daytime RFB for 2 days could decrease sleep latency, the number of awakenings, and awakening time during sleep, and increase sleep efficiency (29). In contrast, it is assumed that using electronic devices at bedtime could be related to shorter sleeping time, extension of sleep latency, and increased sleep deprivation (30). Therefore, using an unfamiliar electronic device might have influenced sleep latency in our study.

The following items can be described as limitations of the present study. First, we did not objectively evaluate sleep function. There are specific measuring devices available for quantitative sleep parameters at home, such as a three-dimensional acceleration device. Using such devices might have further clarified the influence of HRV-BF on sleep. Furthermore, a previous report indicated that HRV-BF before bedtime increased respiratory sinus arrhythmia during sleep (12), but this could not be evaluated in our study because there was no HRV measurement during sleep. Second, this study population consisted of family caregivers of patients with cancer, which made it ethically difficult to include a control group. Therefore, we could not conclude a positive effect on sleep with two sessions of HRV-BF. Since both groups each showed significant improvements, we expected there would be a difference in a group comparison between these groups and a control group. Third, we did not accomplish a long-term evaluation. We found that the HRV-BF sessions produced an effect on the sleep of family caregivers in the medium- to short-term, but did not determine whether such an effect could become persistent. In addition, our follow-up survey indicated that the wearable device at home resulted in insufficient health management in the use group compared with the non-use group, because of a sense of security from using the device (31). Last, because there was a versatility issue because of the single-center design, larger-scale data are required in further studies.

## Conclusions

Our study suggests that two sessions of HRV-BF could be useful to enhance sleep and autonomic nervous function among family caregivers of patients with cancer. Furthermore, additional actual practice of RFB with use of a portable device at home before bedtime may enhance the effect.

## Data Availability Statement

All datasets generated for this study are included in the article/supplementary material.

## Ethics Statement

The studies involving human participants were reviewed and approved by the ethics committee of Kansai Medical University, Japan (No. 2015660). The patients/participants provided their written informed consent to participate in this study.

## Author Contributions

HH was responsible for the conception and design of this study and wrote the article, which was critically revised by all the other authors. HH and MF were responsible for data collection and for clinical evaluations. HH and KK were responsible for data analysis. All authors have approved the final version of this manuscript.

### Conflict of Interest

The authors declare that the research was conducted in the absence of any commercial or financial relationships that could be construed as a potential conflict of interest.

## Abbreviations

HRV-BF: heart rate variability biofeedback
RFB: resonant frequency breathing
PSQI: Pittsburgh Sleep Quality Index
QOL: quality of life
HRV: heart rate variability
BF: biofeedback
J-ZBI: Japanese version of the Zarit Caregiver Burden Interview
LF: low frequency
HF: high frequency
SDNN: standard deviation of the normal-to-normal interval
ANOVA: analysis of variance.
